# Dynamics of Growth and Egg Traits in Three Dietary Balanced Protein Scenarios Applied for Laying Hens

**DOI:** 10.3390/ani12111371

**Published:** 2022-05-27

**Authors:** Ingryd Palloma Teodósio da Nóbrega, Matheus de Paula Reis, Freddy Alexander Horna Morillo, Luis Filipe Villas-Bôas de Freitas, Letícia Cardoso Bittencourt, João Batista Kochenborger Fernandes, Nilva Kazue Sakomura

**Affiliations:** 1 Department of Animal Sciences, Faculty of Agrarian and Veterinary Science, São Paulo State University, Jaboticabal 14884-900, SP, Brazil; palloma_nobrega@hotmail.com (I.P.T.d.N.); matheusdpreis@gmail.com (M.d.P.R.); horna_fr21@hotmail.com (F.A.H.M.); luisfilipevbf95@gmail.com (L.F.V.-B.d.F.); jb.fernandes@unesp.br (J.B.K.F.); 2 DSM Brazil, Mairinque 18120-000, SP, Brazil; leticia.cardoso@dsm.com

**Keywords:** essential amino acid, body components, egg, poultry

## Abstract

**Simple Summary:**

This study aimed to investigate the impact of three dietary balanced protein levels on laying hens, during the rearing and laying phases. The performance and body composition were monitored at 7, 11, 15, and 18 weeks old, while for the laying phase the responses were monitored every 28 days, from 19–102 weeks of age. The dietary protein applied in this study did not affect the responses evaluated in the rearing phase, but it clearly affected the long-term egg production. Overall, the egg production of hens consuming a high protein diet was superior compared to hens in the lower protein group and similar results were observed for body weight and body composition. The benefits of this study were to demonstrate the dynamic traits of laying hens in the long-term egg production cycle in three dietary balanced protein scenarios as similar data could not be found elsewhere.

**Abstract:**

The objective of this study was to evaluate laying hens from 8 to 102 weeks old, regarding their changes in performance, body composition, and egg components produced in three scenarios of nutrition. Three treatments designed to contain different levels of balanced protein (BP) were randomly assigned to the experimental units, performing ten replicates per treatment with 20 birds each. A standard feed was formulated to meet hen requirements and the ideal ratio between essential amino acids. Then, two experimental feeds were formulated to contain 20% above or below the dietary BP used in the standard feed. The responses evaluated were cumulated feed intake (g), daily feed intake (g/day), body weight (g), body composition (g of protein, fat, and ash), hen-housed egg production (%/hen-housed), egg production (%), egg weight (g), egg mass (g), and egg components (percentages of yolk, albumen, and eggshell). The dietary BP influenced the body composition, egg production, egg weight, and egg mass of white laying hens. The increase in dietary BP was related to an increase in body contents and egg weight, whereas hens consuming the low dietary balanced protein presented a lower body weight, leaner, and produced smaller eggs.

## 1. Introduction

Currently, there is a concept to keep hens for extended periods in production, aiming to increase profitability and sustainability. Despite the benefits of doing so, controlling the excess body fat and eggshell quality in old laying hens is reported to be the main concern on poultry farms [1,2]. The body fat and egg components are influenced by the feed offered [3] and eggshell quality reduces as egg weight increases, which may be partially controlled with nutritional strategies [4,5]. In addition, the feed offered to hens during the rearing phase may affect the development of reproductive organs [6], influencing the long-term laying cycle.

The ability of laying hens to overcome a nutritional deficiency or an imbalanced diet is not completely elucidated. Some effort was made to investigate the effects of a previous feed on the laying cycle phase [7,8,9] but little or no knowledge is available in the literature describing how a modern hen may deal with a deficient diet during the growth and its impact in a long-term laying cycle. This information is convenient for poultry nutritionists because they often change the feed formula to improve the economic return and sustainability of egg production farms. In this sense, protein is frequently investigated in poultry nutrition given its importance for growth [10,11], egg production [12,13], economic return [14,15], and sustainability of the farm [16,17]. Because amino acids are the basic constituents of proteins and that essential amino acids should be offered in the feed in a proper ratio with lysine [18], it seems reasonable to investigate the effects of balanced protein in a long-term egg production cycle.

Understanding the dynamics of body and egg components represents a step towards an effective way to improve long-term egg production given feeds should be formulated based on physiological needs and the response of laying hens. In a conventional poultry house, the feed offered is the only source of energy and nutrients for a laying hen. Therefore, a change in voluntary feed intake is the only mechanism that a hen can use to consume a proper amount of all nutrients. If they fail to do so, body and egg components are expected to change [6,19] along with the egg production [20]. This highlights the importance to elucidate the dynamics of body and egg composition over different sets of nutrition scenarios.

The effects of dietary balanced protein for hens in the rearing phase and its cumulated influence in the long-term laying cycle have not been investigated so far. In the context aforementioned, we hypothesized that balanced protein levels affect the body and egg components leading to a shift in the long-term laying cycle; thus, the aim of the present research was to describe how laying hens respond to three levels of dietary balanced protein from 8 to 102 weeks old.

## 2. Materials and Methods

### 2.1. Ethics Approval

All procedures described were approved by the Ethical Committee on the Use of Animals of the School of Agrarian and Veterinary Sciences, São Paulo State University (UNESP), Jaboticabal, São Paulo, Brazil (Process 012598/2018; approved on 14 February 2019).

### 2.2. Bird Husbandry

A total of 600 Lohmann LITE LSL-NA were obtained from a breeding company (Planalto Postura LTDA. Uberlândia, MG. Brazil). The hatchlings were raised in conventional cages from 1 to 7 weeks before being moved to wire rearing cages (375 cm^2^ per pullet) from 8 to 18 weeks. At 19 weeks, hens were transferred to wire-laying cages (563 cm^2^ per hen). All cages were equipped with trough feeders and nipple drinkers. Hens received a corn-soybean meal-based diet to meet or exceed breeding company recommendations from 1 to 7 weeks old. A feed program with three feeds was offered from 8 to 18 weeks, for grower (8–11 weeks), developer (12–15 weeks), and pre-laying phases (16–18 weeks). At the laying phase, a feed program with five feeds was formulated according to the breeding company recommendations: layer one (19–26 w-old), layer two (27–46 w-old), layer three (47–66 w-old), layer four (67–82 w-old), and layer five (83–102 w-old). Birds had free access to feed and fresh water throughout the trial. The lighting program adopted was 24 L in the first week, reduced gradually to 12L:12D up to 10-w-old, which was maintained until the pullets achieved 5% of egg production (20-w-old). After the onset of egg production, the lighting program was gradually increased from 12–16 h of light by adding one hour per week and was then kept constant up to 102 weeks of age. In the period of 0–7, 8–18, and 19–102 w-old, the maximum temperatures recorded were 31, 26, and 27 °C, while the minimum were 24, 17, and 19 °C, respectively. The maximum relative humidity of the air was 77, 79, and 84% while the minimum records were 51, 57, and 50%, respectively for the same phases. One laying cycle was considered as 28 consecutive days.

### 2.3. Experimental Design and Feeds

Three treatments were randomly assigned to 30 experimental units of 20 pullets each, totalling ten replicates per treatment, performing a completed randomized design. Treatments consisted of three dietary levels of balanced protein (BP): 1—standard feed (S), formulated to meet or exceed breeding company recommendations; 2—reduction of 20% in dietary balanced protein (L), in reference to the S feed; 3—increase of 20% in dietary balanced protein (H), in reference to the S feed. Dietary balanced protein was defined as a constant ratio of essential amino acids with lysine [21] and the ratio was the same proposed by the breeding company [22]. Standardized ileal digestible lysine (SID-Lys) was the reference to produce the three levels of dietary balanced protein (Table 1).

In the rearing phase, the S-BP group consumed a feed containing 0.80, 0.70, and 0.74 % of SID-Lys for the grower, developer, and pre-layer phase, respectively. The S-BP feeds in the laying phase contained 0.68, 0.66, 0.63, 0.61, and 0.58% of SID-Lys, respectively for each layer phase (Table 2). The remaining nutrients and energy in the feed were as recommended by the guideline [22].

### 2.4. Performance Data and Egg Components Measurement

The number of eggs produced, and mortality were daily recorded. Every week, all eggs produced were weighed and the egg mass (rate of egg produced × egg weight) was calculated. The feed leftovers were weighed fortnightly and adjusted by mortality to calculate the food intake. The cumulative feed intake was expressed on g/bird for the rearing (eight to 18 w-old) and the rearing plus laying phases (8–102 w-old). Hen-housed egg production was calculated based on the number of eggs produced in the entire experiment period per number of housed hens at 19 w-old.

### 2.5. Body Composition

On the first day of the trial, eight birds per treatment were randomly selected and identified for body composition measurements by dual-energy X-ray absorptiometry (Hologic-QDR^®^ model 13.4.2., Marlborough, MA, USA). Throughout the experiment, the same hen was scanned on the last day of every feeding phase. Prior to each scan, hens were fasted for five hours, weighed, anesthetized with isoflurane (2%) diluted in 100% of oxygen, and positioned in dorsal decubitus with the wings and legs flexed [23]. Measures collected were fat mass (g), lean mass (water + protein content, g), bone mineral content (g), and bone mineral density (g/cm²). Data collected were converted to contents of body protein, fat, and ash by applying the equations published by Alves et al. [23].

### 2.6. Egg Components

At the end of each laying cycle (28 days), a total of nine eggs per experimental unit were collected (three eggs per three sequential days). On each day, the eggs were broken apart individually to measure the albumen, yolk, and shell weights. Before measurement, the eggshell was washed with tap water and dried using a forced oven at 55 °C for 24 h. The percentages of albumen, yolk, and eggshell were then calculated.

### 2.7. Statistical Analysis

The collected data were examined for outliers, normality, and homoscedasticity. The data of cumulative feed intake and hen-housed egg production were analysed as One-Way ANOVA with a Tukey test to evaluate the differences between dietary balanced protein levels, using a generalized linear model. Two-factor repeated measure design was employed to determine the effects of dietary balanced protein over time, using a mixed model. One factor is represented by the three groups receiving the different series of dietary balanced protein feeds and the other factor is the age of hens. Each experimental unit was the repeated measures factor. Differences were considered to be significant at a probability of 5%. The Statistical Analysis System (SAS Institute Inc., Cary, NC, USA) was used to perform both a One-Way ANOVA and the Two-factor repeated measure analyses procedures. The data was analysed considering 21 cycles of four weeks each.

To test whether the responses differed between dietary balanced protein levels over time, non-linear regression with groups was used, the groups being the dietary balanced protein [24]. The average data per replicate were treated as the experimental unit. Two exponential models were applied and that with the lower Akaike information criterion [25] value was used to describe the response variable. The model used were:

(1) $$\mathrm{Linear}\;\mathrm{plus}\;\mathrm{exponential}:\;y=A+B\times \left(R^{age}\right)+C\times age$$

where *A* and *C* are the y-intercept and slope of the linear segment, respectively, *B* is the y-intercept of the exponential segment, and *R* is the exponential base.

(2) $$\mathrm{Exponential}:\;y=A1+B1\times \left(R1^{age}\right)$$

where *A*1 + *B*1 is the y-intercept, and *R*1 is the exponential base.

## 3. Results

The reduction and increase of dietary balanced protein in the laying feed did not affect the cumulative feed intake (*p* > 0.05, Table 3), being on average 4.44 kg (*p* = 0.986) and 67.7 kg of feed per bird (*p* = 0.485) in the growth and whole period, respectively. The reduction of dietary balanced protein affected the hen-housed egg production (*p* < 0.01), with similar results between hens from the S and H groups (*p* > 0.05).

There was an interaction between dietary balanced protein and hens age for feed intake, egg production, and egg mass (*p* < 0.05, Table 4). For feed intake, differences between treatments were observed only at 26 weeks of age (*p* < 0.05). For egg production, differences were observed mainly at the beginning (first three laying cycles) and the end (after 74 w-old) of the laying cycle, whereas for egg mass the differences between groups of hens were consistent during the whole experimental period (Table 4).

**Figure 1:**
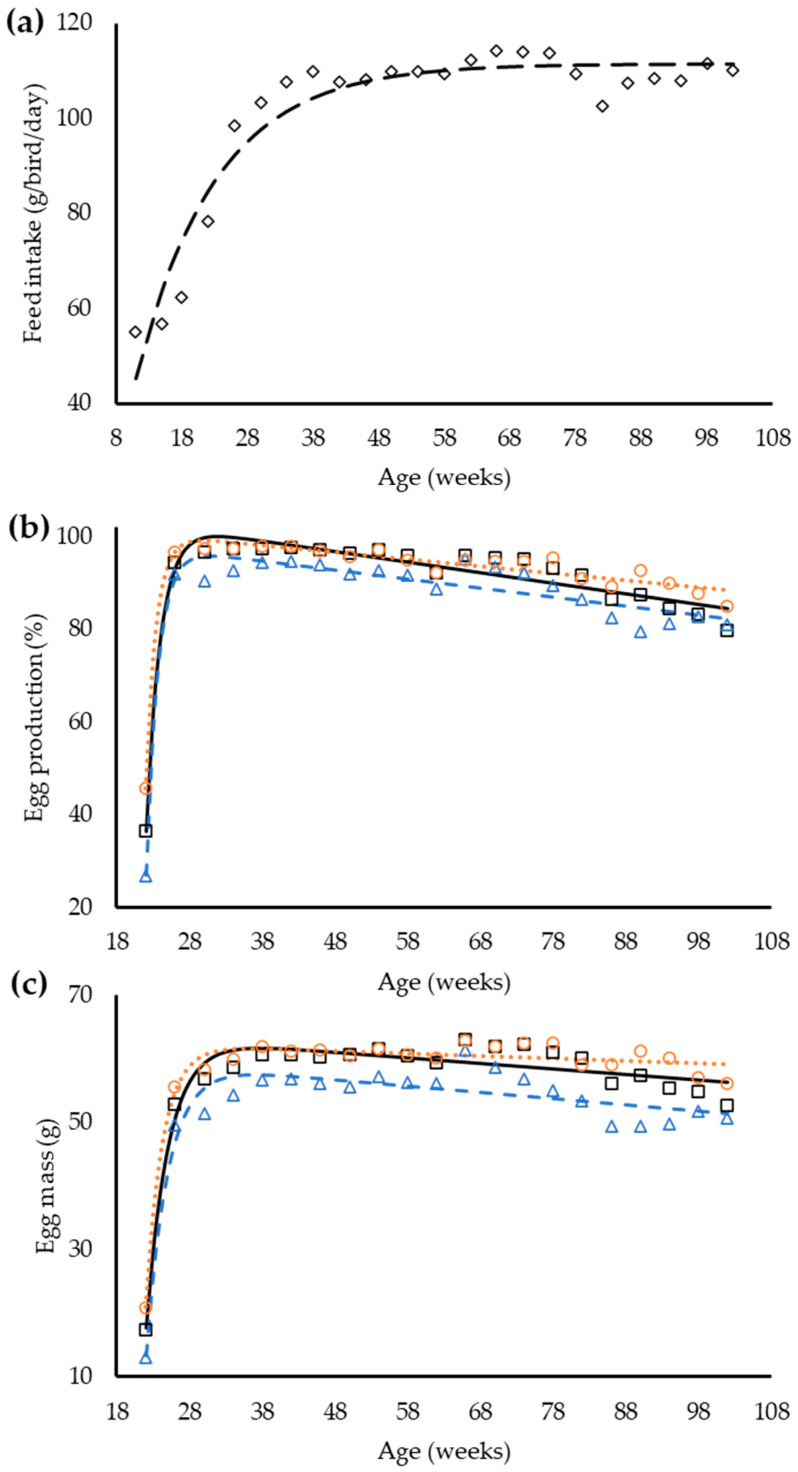
Observed and predicted feed intake ((**a**) ◊, – –) of laying hens from 8 to 102 weeks old and of egg production (**b**) and egg mass (**c**) of laying hens from 19 to 102 weeks old in response to age in three dietary balanced protein levels: standard (□, —), low (∆, - -), and high (○, ∙∙∙).

The exponential equation was used to demonstrate the changes in feed intake and the line plus exponential equation had the best fit for egg production and egg mass (Table 5). The regression with groups identified that a single equation could be used to describe the feed intake between groups, whereas for egg production and egg mass the regression analysis indicates a necessity for different equations for laying hens inside each dietary balanced protein group (Figure 1).

Differences in body weight influenced by dietary balanced protein were observed in 30 w-old hens (*p* < 0.05, Table 6), with heavier hens in the H group, followed by the S and L groups, respectively. Laying hens in the higher dietary balanced protein feed was fatter (*p* < 0.05) from 38 w-old and forward (Table 6).

Body contents of ash and protein increased along time for all groups (*p* < 0.05, Table 7). For body ash, differences were observed from 50 w-old and body protein from 30 w-old, and still relatively constant until the end of the trial.

**Figure 2:**
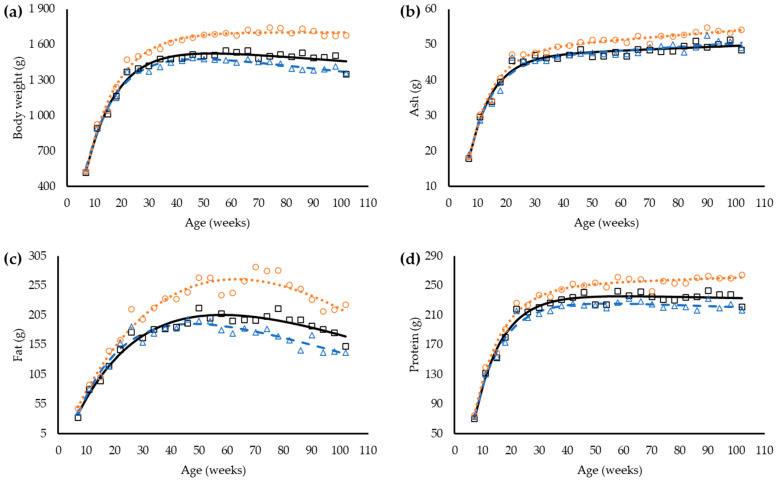
Observed and predicted body weight (**a**) and body components (ash (**b**), fat (**c**), and protein (**d**)) of laying hens from 8 to 102 weeks old fed three balanced protein feeds: standard (□, —); low (∆, - -) and high (○, ∙∙∙).

The linear plus exponential function had the best fit for body weight and body components, and it was used to investigate the differences between hens consuming the different feeds (Table 8). A common coefficient R-value can be used to describe the changes in growth (*p* < 0.05), despite the levels of dietary balanced protein, except for fat (*p* < 0.05). However, the coefficients A, B, and C are different between groups (*p* < 0.05) and specific values are necessary to properly describe the changes in body weight and body composition between hens consuming the different levels of dietary balanced protein (Figure 2).

Overall, egg weight of laying hens was affected by dietary balance protein content (*p* < 0.05, Table 9). At 26 w-old, hens consuming the H feed produced heavier eggs, followed by hens from the S and L groups, respectively. Notably, egg weight was similar between groups of hens from 30 to 42 w-old and from 54 to 66 w-old (*p* < 0.05). The yolk percentage increased in all feed treatments as the hens aged (*p* < 0.05).

Different from those observed for yolk, the albumen and eggshell percentages reduced with time (*p* < 0.05, Table 10). Nevertheless, egg components were similar between groups of hens (*p* > 0.05) with a tendency for yolk percentage (*p* < 0.06).

**Figure 3:**
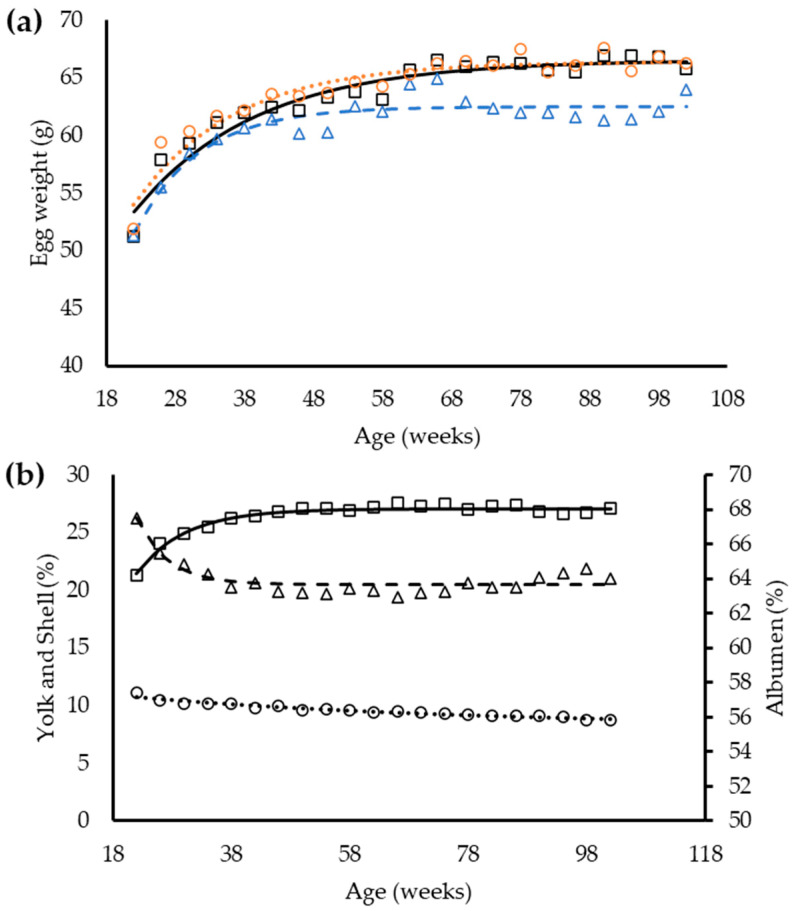
Observed and predicted egg weight (**a**) of laying hens from 19 to 102 weeks old in response to age in three dietary balanced protein levels (standard (□, —); low (∆, - -), and high (○, ∙∙∙)), and egg components (**b**) yolk (□, —), albumen (∆, - -) and shell (○, ∙∙∙).

The exponential function had the best fit for egg weight and egg components (Table 11). The analysis indicates that all dietary balanced protein contents used in this study affected the egg weight (*p* < 0.05) and all equation coefficients need to be changed to estimate the egg weight of hens according to dietary balanced protein. The range in dietary balanced protein levels applied in this study was not sufficient to change the concentration in egg components (*p* < 0.05); therefore, a single exponential equation was used for each egg component. A tendency was observed for yolk percentage (*p* = 0.06), suggesting an influence of dietary balanced protein in this egg component. The equation used to describe the albumen percentage had a low R^2^ value, mainly because a drop in albumen percentage was observed around 68 w-old and followed by a consecutive increase (Figure 3), which was poorly predicted with the exponential equation used.

## 4. Discussion

The aim of the study was to describe how three scenarios of protein levels elicited variations in the growth of laying hens and how such changes might affect long-term egg production and egg components. To our knowledge, this is the first study to investigate the influence of dietary balanced protein in laying hens, from the rearing period (eight w-old) until the end of laying cycle (102 w-old). Currently, there is a growing concept to keep laying hens for longer periods in production [1]. However, maintaining the egg production and egg quality of a flock of older hens is a challenge. Pieces of evidence demonstrate that body weight of laying hens at the onset of lay may affect the entire egg production [26] and the egg weight [27,28]. Specifically, the body composition of laying hens at the beginning of the laying phase could also affect the peak and persistence of egg production [29]. The dietary protein content is known to affect the growth of broilers and breeder pullets [30,31], and considering the higher cost of dietary protein [32] and the trend in reducing the nitrogen excretion in poultry farms [17], it might be convenient to investigate the effects of dietary protein over the growing and laying phase. Assuming that essential amino acids are required in constant ratios with lysine, in this study the concept of balanced protein was used as proposed by Eits et al. [33].

We observed that dietary balanced protein levels used in this study, poorly affected the daily feed intake of laying hens. A general theory for feed intake regulation was developed over the years [34,35,36], suggesting that feed intake is regulated by the first limiting component in the feed, being energy or essential amino acids. Evidence demonstrates that feed intake of growing broiler chicken and pullet of broiler breeder is affected by dietary protein [30,31]. For laying birds, the feed intake regulation seems to be more complex because the consumed nutrients are also used for egg production. A model proposed by Fisher et al. [37] and recently reviewed by Sakomura et al. [38] accommodated this problem, splitting the amino acid requirement for maintenance and egg mass, which was called the Reading model. Those authors introduced a methodology to predict the requirements of essential amino acids (mg/hen/day), highlighting the importance to understand the mechanisms related to feed intake regulation. In the present study, the higher level of dietary balanced protein elicited an increase in egg mass. According to the cited model, egg mass will affect the requirement of essential amino acids, which may explain why feed intake did not reduce for laying hens in the H group. On the contrary, laying hens in the L group reduced the egg mass, therefore, a lower amino acid was needed for egg production, which may have impacted the feed intake. An interesting behaviour of feed intake was observed after 74 weeks. Laying hens from all groups reduced their intake of feed. For egg production, comparing the values in the peak and that observed at week 74th, egg production reduced by about 2.6, 2.5, and 3.2 units, respectively for hens consuming the standard, low, and high dietary balanced protein feeds, which may be related to a reduction in laying hens needs, consequently, the feed intake.

On the other hand, when the cumulated feed intake was calculated per unit of egg produced, it was evidenced that laying hens in the L group consumed 130 g of feed per unit of egg produced, whereas the S and H groups consumed 122 and 120 g of feed per egg produced. The feed intake per unit of body weight was 50.3, 49.7, and 44.2 kg of feed per kg of body weight for the L, S, and H groups, respectively. Those results suggest that hens attempted to regulate their feed intake to compensate for the reduction in dietary balanced protein when the feed is deficient and reduce the feed intake when the dietary balanced protein is in excess. Recently, Kumar et al. [39] described a quadratic response of feed intake in function of the dietary balanced protein. Different from the study reported herein, laying hens (Lohmann-LSL) received a standard feed in the rearing phase. The laying hen’s current status, regarding body weight and body composition, seems to be an important factor that modulates their response and needs more attention in future studies.

Even though the reduction in dietary balanced protein may reduce the feed cost and nitrogen excretion [16,40], the feed intake per hen-housed egg increased. In this study, the number of eggs produced per hen-housed reduced 31 units for hens in the L group compared with the S group. Therefore, feeding cost (feed price x feed intake), revenue, and viability of hens should be accounted for to properly calculate the economic return. Viability observed in the L group was 85%, whereas for the S and H groups were 90 and 93%, respectively. Laying hens consuming the L feed demonstrated an acute reduction in body fat after 54 w-old. The ovulation cycle was demonstrated to be dependent on plasma-free fatty acids and the body lipid seems to be the main blood source of fatty acids [41]. We hypothesized that the ovulation cycle was affected by a reduction in body fat content, which reduces egg production in the L group. Eventually, a severe reduction of body fat might drastically affect the ovulation cycle and may stop egg production, reducing the viability of hens in the L group.

Laying hens from the H group had a body weight close to the recommendations in the guideline (Lohmann Tierzucht GmbH, Cuxhaven, Germany), whereas the hens from the S and L groups were 200 g lighter. The variation in body weight was mostly due to body fat, since after sexual maturity there is a reduction in body protein deposition, and the change in body weight is given by variations in body fat deposition [2,42]. The observations on body fat and egg production suggest that the hens in the H group did not have an excess of body lipid, as the laying performance was not affected. On the contrary, the persistence of egg production indicates that body fat in the H group was favourable. Milisits et al. [29] observed that laying hens with high body fat content at the onset of lay reduced the egg production in about 11 to 13 eggs when compared with hens with lower body fat content. There is a discussion about the importance of energy reserves as body fat; however, there is a lack of information on the desired body fat content that benefits longer-term egg production.

Using the first derivative of the linear plus exponential equation, the results demonstrated an increase in body fat content until 48 (L), 58(S), and 63 (H) w-old, followed by a linear reduction until 102 w-old. As cited before, few differences were observed between groups, where laying hens from the H group seem to have a delay in body fat mobilization, regarding the age. After the cited ages, the laying hens mobilize body fat, possible to maintain egg production, especially after 82 w-old, when the dietary metabolizable energy was reduced, as recommended by the guideline. Nonis and Gous [43] demonstrated that broiler breeders produce energy from body lipid if they are allowed to do it so, even though the concentration of dietary energy is above requirement, by regulating their feed intake. Similarly, Caldas et al. [44] observed a reduction in broiler breeders fat at the end of egg production phase. In line with our findings, Kumar et al. [17] observed a linear increase in body fat for laying hens consuming a feed with a crescent level of digestible lysine (ranging from 560 to 858 mg/hen/day). However, the authors investigated the effect of balanced protein in Lohmann-LSL Lite NA only until 66 w-old.

The body ash content of laying hens suggested that laying hens did not use mineral reserves to produce an egg, as the body ash increased until the end of the laying cycle. The major portion of minerals used for egg formation is due to calcium carbonate necessary for eggshell formation given approximately 80% of eggshell is formed by this mineral [45]. Around 99% of total body calcium is found in the bone ash [46]. Evidence demonstrates that in a flock of older hens, there are individuals with a tendency to develop osteoporosis [47], and there are an increased number of eggs with thinner eggshells due to lower ability to uptake calcium and phosphorus from the intestinal lumen [48]. In this study, dietary balanced protein seems to have a low or no effect over the dynamics of body ash contents. Apparently, the advanced ages of laying hens used in this study was not sufficient to elicit a negative consequence in the bone structure; however, in all treatments, the eggshell percentage reduced with age, probably due to the increase in egg size with the age of laying hens [49].

The dynamic of egg components observed over time was similar to that reported by Bendezu et al. [5] for white laying hens from 18 to 60 w-old. As the laying hens aged, the yolk percentage increased, and the albumen and eggshell reduced, which was consistent with other reported literature [50,51]. The dietary balanced protein tended to influence yolk percentage. Compared with the L group, the hens consuming the H feed produced eggs with more percentage of yolk. The contribution of dietary protein to yolk formation is probably related to phosvitin as this is the major protein molecule found in egg yolk. Around 56% of the amino acid found in the phosvitin is serine phosphorylated [52,53]. According to Huang et al. [54], it is believed that the role of phosvitin in the egg is related to embryo development, which reinforces the importance of such a constituent in egg yolk. In the present study, the tendency of a lower percentage of yolk observed in eggs of the L group may be related to the lower amount of dietary serine, which is necessary to produce the phosvitin in the egg. With lower sources of dietary serine, essential amino acids might be used to overcome this deficiency.

Among the responses observed in this study, the body fat content between laying hens consuming the different levels of balanced protein was unexpected. Most reports in the literature demonstrate that growing birds would increase body lipid content when they are offered a low balanced protein feed [30,31]. The opposite result is reported when a growing bird consumes a high balanced protein feed. The regulation of body fat content in laying birds seems to be more complex and the prediction of body fat in laying hens should be done with caution. The increase in egg yolk percentage may contribute to a higher value of body fat content as 34% of egg yolk is constituted of lipid [55]. Hocking [56] investigated the effect of body weight and feed intake on the ovarium follicular dynamics and found that feed-restricted broiler breeders reduced the number of yellow follicles. We did not find a similar study for laying hens, which would contribute to a better understanding of the results observed, but we hypothesize that a pullet raised with high level of dietary balanced protein feed may increase the number and the weight of yellow follicles in the ovarium, resulting in more body fat content in laying hens.

## 5. Conclusions

As expected, the dietary balanced protein influenced the dynamics of performance, body content, egg production, and egg mass of laying hens in the laying phase. The performance of laying hens increased with higher levels of balanced protein but other responses such as the feeding cost also influence the economic return and need to be considered to making a nutritional decision. The hen-housed egg production reduced in laying hens consuming a feed with low levels of dietary balanced protein. In this study, body ash was not mobilized, indicating that the minerals consumed were sufficient for egg production and that the dietary balanced protein levels applied in this study did not influence this variable. On the contrary, a mobilization of body fat was observed, being more evident at the end of laying cycle. The dietary balanced protein levels investigated in this study slightly affected the yolk percentage but had no influence on albumen and eggshell percentages. More persistence of egg production was observed for laying hens consuming a high dietary balanced protein feed.

## Figures and Tables

**Table 1:** Composition and nutritional content of experimental feeds in the rearing phase.

Composition (%)	Grower (8–11 Weeks)			Developer (12–15 Weeks)			Pre-Layer (16–18 Weeks)		
	Standard	Low	High	Standard	Low	High	Standard	Low	High
Corn (8.8%)	62.2	68.0	56.3	58.2	63.0	53.4	55.5	61.0	49.9
Soybean meal (45%)	23.3	15.0	31.6	16.9	10.0	23.8	17.1	10.0	24.2
Wheat bran	10.1	13.0	7.10	17.5	20.0	15.0	17.0	20.0	14.0
Potassium carbonate	0.120	0.240	-	0.105	0.210	-	0.143	0.280	0.005
Corn gluten (60%)	-	-	-	1.50	1.50	1.50	1.00	-	2.00
Meat and Bone Meal 48%	-	-	-	2.66	2.66	2.66	3.42	2.97	3.87
Soybean oil	0.825	0.150	1.50	0.935	0.370	1.50	1.05	0.685	1.42
Dicalcium phosphate	1.18	1.17	1.20	0.150	0.140	0.161	0.105	0.210	-
Limestone	1.39	1.46	1.32	1.32	1.38	1.26	3.87	3.98	3.75
Salt	0.353	0.287	0.420	0.260	0.215	0.306	0.236	0.215	0.256
Sodium Bicarbonate	0.100	0.200	-	0.133	0.200	0.065	0.154	0.198	0.110
Vitamin and Mineral Premix ^1^	0.200	0.200	0.200	0.200	0.200	0.200	0.200	0.200	0.200
DL-Methionine (99%)	0.108	0.055	0.161	0.057	-	0.114	0.078	0.045	0.111
L-Lysine HCl (78%)	0.062	0.100	0.024	0.019	0.038	-	0.061	0.095	0.027
L-Threonine (98.5%)	0.015	-	0.031	-	-	-	-	-	-
Choline chloride (60%)	0.100	0.100	0.100	0.100	0.100	0.100	0.100	0.100	0.100
Total	100	100	100	100	100	100	100	100	100
**---------- Calculated nutritional content (%) ----------**									
Met. energy (kcal/kg) ^2^	2881	2883	2880	2860	2860	2860	2778	2778	2778
Crude protein ^3^	17.1	14.3	20.0	17.1	14.7	19.5	17.0	13.9	20.2
Crude fibre ^3^	3.22	3.28	3.30	4.32	4.15	3.75	4.42	3.96	4.33
Starch ^3^	45.9	46.8	43.0	40.2	43.1	41.5	38.9	43.3	37.8
Digestible Lysine	0.803	0.645	0.960	0.700	0.560	0.840	0.742	0.593	0.890
Digestible Methionine + cysteine	0.592	0.476	0.709	0.544	0.434	0.653	0.552	0.442	0.662
Digestible Threonine	0.589	0.475	0.703	0.553	0.471	0.635	0.546	0.441	0.652
Digestible Tryptophan	0.187	0.148	0.226	0.171	0.139	0.203	0.169	0.133	0.206
Digestible Isoleucine	0.628	0.496	0.760	0.589	0.480	0.698	0.581	0.444	0.719
Digestible Valine	0.698	0.568	0.827	0.680	0.573	0.787	0.672	0.532	0.812
total Calcium	1.04	1.04	1.04	1.05	1.05	1.05	2.08	2.08	2.08
Available Phosphorus	0.460	0.460	0.460	0.430	0.430	0.430	0.457	0.457	0.457
Sodium	0.180	0.180	0.180	0.170	0.170	0.170	0.170	0.170	0.170

^1^ Content/kg of feed: Vit. A 9700 IU, Vit. D3 2700 UI, Vit. E 17 UI, Vit. K3 2.79 mg, Vit. B1 2.00 mg, Vit. B2 5.50 mg, Pantothenic acid 10.6 mg, Vit. B6 3.05 mg, Vit. B12 15.0 mcg, Niacin 0.039 g, Folic acid 1.00 mg, Biotin 0.0083 mg, Choline chloride 0.150 g, Iron 0.044 g, Copper 9.00 mg, Manganese 0.050 g, Zinc 0.050 g, iodine 1.00 mg, selenium 0.250 mg, Phytase 600 FYT. ^2^ Nitrogen-corrected apparent metabolizable energy. ^3^ Values represent the mean analysed composition by near-infrared spectroscopy (NIR).

**Table 2:** Composition and nutritional content of experimental feeds in the laying phase.

Composition (%)	Layer 1 (19–26 Weeks)			Layer 2 (27–46 Weeks)			Layer 3 (47–66 Weeks)			Layer 4 (67–82 Weeks)			Layer 5 (83–102 Weeks)		
	Standard	Low	High	Standard	Low	High	Standard	Low	High	Standard	Low	High	Standard	Low	High
Corn (8.8%)	59.0	65.0	53.0	61.1	67.0	55.3	61.9	67.5	56.2	63.3	68.5	58.0	63.4	68.7	58.1
Soybean meal (45%)	16.1	10.1	22.0	18.4	13.0	23.9	16.9	11.6	22.3	16.6	11.7	21.4	16.1	11.9	20.2
Wheat bran	3.75	6.00	1.49	2.35	4.70	-	2.50	5.00	-	2.50	5.00	-	2.50	5.00	-
Potassium carbonate	0.450	0.560	0.340	0.386	0.470	0.302	0.425	0.525	0.325	0.408	0.500	0.315	0.425	0.510	0.340
Corn gluten (60%)	7.50	5.00	10.0	5.43	2.55	8.30	5.76	2.95	8.56	5.01	1.95	8.07	4.54	1.10	7.98
Soybean oil	1.07	0.890	1.25	0.725	0.610	0.840	0.680	0.570	0.790	0.566	0.580	0.552	0.670	0.660	0.680
Dicalcium phosphate	1.31	1.30	1.32	1.16	1.14	1.17	1.11	1.09	1.14	1.11	1.09	1.14	1.01	0.98	1.04
Limestone	9.11	9.17	9.06	9.46	9.51	9.41	9.77	9.82	9.72	9.77	9.82	9.72	10.4	10.4	10.3
Salt	0.307	0.279	0.336	0.323	0.290	0.356	0.292	0.275	0.310	0.270	0.260	0.280	0.275	0.280	0.270
Sodium Bicarbonate	0.155	0.200	0.110	0.132	0.183	0.080	0.139	0.168	0.110	0.174	0.190	0.157	0.166	0.160	0.172
Vitamin and Mineral Premix ^1^	0.200	0.200	0.200	0.200	0.200	0.200	0.200	0.200	0.200	0.200	0.200	0.200	0.200	0.200	0.200
DL-Methionine (99%)	0.066	0.041	0.090	0.065	0.047	0.082	0.052	0.036	0.068	0.048	0.038	0.059	0.044	0.038	0.050
L-Lysine HCl (78%)	0.099	0.119	0.078	0.015	0.029	-	0.019	0.038	-	0.011	0.022	-	-	-	-
Choline chloride (60%)	0.100	0.100	0.100	0.100	0.100	0.100	0.100	0.100	0.100	0.100	0.100	0.100	0.100	0.100	0.100
Inert ^2^	0.822	1.023	0.621	0.075	0.150	-	0.165	0.159	0.171	0.026	0.052	-	0.248	-	0.495
Total	100	100	100	100	100	100	100	100	100	100	100	100	100	100	100
**---------- Calculated nutritional content (%) ----------**															
AMEn (kcal/kg) ^3^	2795	2795	2795	2785	2785	2785	2785	2785	2785	2785	2785	2785	2770	2770	2770
Crude protein ^4^	18.1	15.6	21.4	17.0	14.6	20.6	16.1	13.3	20.2	16.0	11.7	18.7	14.8	11.4	18.7
Crude Fibre ^4^	3.68	3.97	3.92	3.66	3.67	3.91	3.65	3.81	3.9	3.57	3.18	3.69	3.46	3.49	3.50
Starch ^4^	40.0	41.3	39.3	39.7	41.3	37.3	41.3	42.4	37.4	40.9	43.4	39.0	43.0	43.2	40.7
Digestible Lysine	0.680	0.544	0.816	0.655	0.524	0.786	0.625	0.500	0.750	0.605	0.484	0.726	0.580	0.464	0.696
Digestible Methionine + Cystine	0.600	0.480	0.720	0.580	0.464	0.696	0.560	0.448	0.672	0.540	0.432	0.648	0.520	0.416	0.624
Digestible Threonine	0.571	0.459	0.683	0.570	0.459	0.681	0.555	0.446	0.664	0.540	0.433	0.647	0.524	0.420	0.627
Digestible Tryptophan	0.157	0.123	0.192	0.163	0.131	0.196	0.156	0.124	0.188	0.153	0.123	0.183	0.148	0.121	0.176
Digestible Isoleucine	0.633	0.486	0.781	0.630	0.484	0.776	0.612	0.468	0.755	0.590	0.450	0.731	0.570	0.434	0.707
Digestible Valine	0.716	0.563	0.869	0.706	0.553	0.859	0.689	0.539	0.839	0.666	0.518	0.813	0.644	0.499	0.789
total Calcium	3.95	3.95	3.95	4.05	4.05	4.05	4.15	4.15	4.15	4.15	4.15	4.15	4.35	4.35	4.35
Available Phosphorus	0.440	0.440	0.440	0.410	0.410	0.410	0.400	0.400	0.400	0.400	0.400	0.400	0.380	0.380	0.380
Sodium	0.175	0.175	0.175	0.175	0.175	0.175	0.165	0.165	0.165	0.165	0.165	0.165	0.165	0.165	0.165

^1^ Content/kg of feed: Vit. A 9700 Ul, Vit. D3 2700 Ul, Vit. E 15.8 Ul, Vit. K3 2.39 mg, Vit. B1 2.40 mg, Vit. B2 6.00 mg, Pantothenic acid 8.65 mg, Vit. B6 3.04 mg, Vit. B12 15.4 mcg, Niacin 0.032 g, Folic acid 1.00 mg, Biotin 0.083 mg, Choline chloride 0.187 g, Iron 0.044 g, Copper 9.00 mg, Manganese 0.050 g, Zinc 0.050 g, iodine 1.00 mg, selenium 0.250 mg, Phytase 600 FYT. ^2^ Inert—Washed sand. ^3^ Nitrogen-corrected apparent metabolizable energy. ^4^ Values represent the mean analysed composition by near-infrared spectroscopy (NIR).

**Table 3:** Cumulative feed intake and hen-housed egg production (± standard deviation) of laying hens from 8 to 102 weeks old in response to age in three dietary balanced protein levels.

Treatments ^1^	Cumulative Feed Intake, kg/Bird		Hen Housed Egg Production ^2^, und
	8 to 18 Weeks	8 to 102 Weeks	
S	4.44 ± 170	67.2 ± 1.82	516 ± 15 ^a^
L	4.45 ± 131	67.4 ± 2.88	485 ± 24 ^b^
H	4.43 ± 151	68.4 ± 1.72	529 ± 22 ^a^
*p*-value	0.986	0.485	0.001

^1^ S—standard, formulated to meet the nutritional recommendation of Lohmann-LSL guideline; L—20% reduction of balanced protein from S; and H—20% increase of balanced protein from S; ^2^ Distinct letter in the same column is significantly different by Tukey’s.

**Table 4:** Performance of laying hens from 8 to 102 weeks old in response to three dietary balanced protein feeds.

Age,	Feed Intake, g/Bird/Day					Egg Production, %					Egg Mass, g				
Weeks	S ^1^	L ^2^	H ^3^	SEM ^4^	*p*-Value	S	L	H	SEM	*p*-Value	S	L	H	SEM	*p*-Value
11	56.0	55.8	53.5	1.16	0.3930	-	-	-	-	-	-	-	-	-	-
15	56.4	56.9	56.8	1.39	0.9740	-	-	-	-	-	-	-	-	-	-
18	61.6	61.8	63.4	1.61	0.7450	-	-	-	-	-	-	-	-	-	-
22	79.2	75.9	80.2	1.26	0.1090	36.6	27.0	45.7	1.51	<0.0001	17.4	13.1	20.9	0.927	<0.0001
26	97.5	95.6	102	1.21	0.0060	94.3	91.9	96.8	1.41	0.0320	52.8	49.7	55.6	0.927	0.0010
30	104	102	103	0.94	0.7870	96.7	90.4	97.5	1.32	<0.0001	56.8	51.4	58.4	0.927	<0.0001
34	109	105	109	1.20	0.1570	97.3	92.6	97.4	1.32	0.0100	58.6	54.3	60	0.994	0.0010
38	109	110	110	1.25	0.7140	97.3	94.4	98.0	1.32	0.0990	60.6	56.7	61.9	0.927	0.0020
42	107	106	110	1.08	0.1850	97.7	94.6	98.0	1.32	0.1140	60.6	56.9	61.3	0.927	0.0060
46	108	109	108	0.78	0.6660	97.3	93.8	96.9	1.32	0.1130	60.4	56.1	61.4	0.927	0.0010
50	109	110	110	1.09	0.8910	96.5	91.9	95.8	1.41	0.0240	60.8	55.7	60.6	0.927	0.0010
54	109	110	111	0.86	0.4860	97.1	92.8	97.3	1.41	0.0220	61.6	57.2	61.6	0.927	0.0040
58	108	110	110	0.92	0.5500	95.8	91.7	94.9	1.51	0.0660	60.6	56.3	60.7	0.995	0.0040
62	111	113	113	1.07	0.6690	92.2	88.7	92.2	1.41	0.0790	59.5	56.1	60.1	0.994	0.0190
66	112	115	115	1.04	0.2590	95.8	95.1	94.9	1.32	0.8650	63.0	61.4	62.9	0.927	0.4860
70	113	115	115	0.900	0.4980	95.4	93.4	94.7	1.51	0.5670	61.9	58.7	62.0	0.995	0.0460
74	113	113	115	1.28	0.5940	95.1	92.1	94.8	1.51	0.2160	62.3	56.8	62.5	0.927	<0.0001
78	109	109	111	1.04	0.5720	93.3	89.5	95.4	1.51	0.0070	61.1	55.0	62.5	0.927	<0.0001
82	101	103	103	1.65	0.7180	91.8	86.4	90.9	1.41	0.0070	60.1	53.5	59.1	0.927	<0.0001
86	107	106	110	1.29	0.1760	86.4	82.5	89.3	1.51	0.0020	56.1	49.5	59.1	0.994	<0.0001
90	109	108	107	1.46	0.6820	87.4	79.6	92.6	1.61	<0.0001	57.4	49.4	61.3	0.994	<0.0001
94	107	109	107	1.73	0.6230	84.5	81.3	89.9	1.41	<0.0001	55.4	49.9	60.2	0.994	<0.0001
98	109	114	111	1.97	0.2150	83.2	82.7	87.7	1.61	0.0190	54.9	51.7	57.1	0.994	0.0020
102	109	111	110	1.90	0.7850	79.9	81.0	85.0	1.41	0.0160	52.7	50.7	56.1	0.995	0.0020
Main effects															
Age					<0.0001					<0.0001					<0.0001
Balanced Protein					0.7210					<0.0001					<0.0001
Interaction					0.0070					<0.0001					<0.0001

^1^ S—standard, formulated to meet the nutritional recommendation of Lohmann-LSL guideline; ^2^ L is low, formulated with 20% reduction of balanced protein from S; ^3^ H is high, formulated with 20% increase of balanced protein from S; ^4^ Standard error of the mean.

**Table 5:** Coefficients from an exponential equation for feed intake of laying hens from 8 to 102 weeks old and coefficients from a linear plus exponential equation for egg production and egg Mass of laying hens from 19 to 102 weeks old in response to age in three dietary balanced protein levels.

Parameters	Feed Intake, g/Bird/Day	Egg Production, %			Egg Mass, g		
		S ^1^	L ^2^	H ^3^	S	L	H
A	111.4	108.0	102.0	104.0	65.30	61.40	63.10
B	−163.1	−5,150,000	−51,900,000	−272,000,000	−63,700	−77,000	−489,000
C	-	−0.2280	−0.1940	−0.1510	−0.0870	−0.0980	−0.0380
R	0.9210	0.5990	0.5410	0.4960	0.7200	0.7140	0.6530
SEM ^4^	6.040	2.920			2.960		
R^2 5^	88.80	95.00			91.00		

^1^ S—standard, formulated to meet the nutritional recommendation of Lohmann-LSL guideline; ^2^ L is low, formulated with 20% reduction of balanced protein from S; ^3^ H is high, formulated with 20% increase of balanced protein from S; ^4^ Standard error of the mean; ^5^ Coefficient of determination.

**Table 6:** Body weight and body lipid of laying hens from 8 to 102 weeks old in response to three dietary balanced protein feeds.

Age,	Body Weight, g					Lipid, g				
Weeks	S ^1^	L ^2^	H ^3^	SEM ^4^	*p*-Value	S	L	H	SEM	*p*-Value
8	518	530	527	8.23	0.9820	38.4	41.1	41.8	1.66	0.8587
11	894	899	926	13.2	0.8608	80.3	81.6	87.7	2.25	0.9586
15	1010	1046	1038	26.1	0.8312	94.6	102	101	4.59	0.9544
18	1152	1148	1229	28.9	0.2692	116	119	139	5.63	0.5461
22	1370	1366	1471	38.5	0.1935	146	154	163	11.2	0.7326
26	1398	1384	1504	46.9	0.1139	176	186	215	19.8	0.3234
30	1422	1374	1533	49.8	0.0349	167	159	198	15.8	0.1676
34	1477	1412	1564	52.5	0.0514	181	174	217	16.5	0.0970
38	1479	1449	1616	46.9	0.0186	170	184	234	11.9	0.0151
42	1485	1466	1641	47.0	0.0092	184	182	232	15.4	0.0351
46	1514	1485	1657	45.2	0.0141	191	198	243	15.7	0.0402
50	1507	1480	1681	46.9	0.0026	205	195	268	14.5	0.0025
54	1523	1472	1689	52.2	0.0011	192	195	268	16.3	0.0014
58	1548	1470	1695	43.7	0.0014	208	180	238	16.1	0.0312
62	1484	1445	1679	52.7	0.0012	183	175	243	17.8	0.0119
66	1550	1479	1724	58.5	0.0004	197	182	263	17.8	0.0006
70	1460	1454	1703	49.2	0.0001	196	176	286	20.2	<0.0001
74	1467	1454	1743	47.3	<0.0001	186	182	280	17.6	<0.0001
78	1483	1441	1740	44.0	<0.0001	213	169	281	18.6	<0.0001
82	1462	1397	1696	41.5	<0.0001	180	162	256	15.3	<0.0001
86	1463	1373	1733	40.4	<0.0001	194	141	250	16.8	<0.0001
90	1452	1357	1714	32.2	<0.0001	184	163	233	19.2	0.0553
94	1477	1374	1672	48.2	<0.0001	172	137	212	14.2	0.0061
98	1501	1398	1675	44.6	0.0002	172	139	214	12.7	0.0079
102	1344	1341	1689	37.1	<0.0001	149	137	229	14.5	0.0051
Main effects										
Age					<0.0001					<0.0001
Balanced Protein					0.0032					0.0147
Interaction					<0.0001					<0.0001

^1^ S—standard, formulated to meet the nutritional recommendation of Lohmann-LSL guideline; ^2^ L is low, formulated with 20% reduction of balanced protein from S; ^3^ H is high, formulated with 20% increase of balanced protein from S; ^4^ Standard error of the mean.

**Table 7:** Body protein and ash of laying hens from 8 to 102 weeks old in response to three dietary balanced protein feeds.

Age,	Protein, g					Ash, g				
Weeks	S ^1^	L ^2^	H ^3^	SEM ^4^	*p*-Value	S	L	H	SEM	*p*-Value
8	73.0	73.2	74.0	1.52	0.9171	18.3	18.9	18.0	0.401	0.8248
11	132	131	140	1.93	0.6790	29.6	28.6	30.3	0.594	0.6182
15	153	154	159	3.87	0.8188	33.9	33.4	34.3	3.89	0.8631
18	178	173	189	5.08	0.4017	39.3	37.1	40.2	0.600	0.1094
22	218	214	227	7.77	0.5049	45.4	45.7	47.2	12.1	0.5528
26	214	206	223	6.50	0.2032	45.2	45.7	47.2	9.23	0.4427
30	220	212	237	7.07	0.0168	47.1	45.4	47.7	11.9	0.3555
34	226	215	235	6.38	0.0651	46.3	45.4	47.7	12.8	0.3993
38	231	223	245	6.61	0.0548	46.1	46.7	49.4	5.00	0.1220
42	234	226	252	6.34	0.0086	47.1	47.1	49.7	9.32	0.1954
46	241	223	250	6.29	0.0096	48.7	47.4	50.6	4.63	0.1569
50	224	224	254	6.72	0.0006	46.5	47.7	51.3	12.9	0.0130
54	227	220	248	6.01	0.0031	47.2	47.3	51.3	11.9	0.0137
58	243	227	262	5.57	0.0004	47.8	47.2	51.3	4.97	0.0331
62	236	232	259	7.02	0.0070	46.8	47.3	50.6	13.5	0.0499
66	242	229	259	7.74	0.0077	48.7	47.7	52.4	13.8	0.0139
70	235	225	242	7.14	0.2201	47.7	49.2	50.2	12.7	0.5501
74	229	220	256	6.67	0.0002	46.8	49.6	52.4	12.8	0.0321
78	223	223	253	6.17	0.0016	47.9	50.0	52.3	14.4	0.0478
82	226	221	253	6.23	0.0017	48.3	47.8	52.7	4.86	0.0121
86	233	213	261	5.68	<0.0001	50.7	48.9	53.6	10.9	0.0431
90	241	228	262	9.90	0.0870	48.1	51.9	54.5	15.4	0.0072
94	235	216	260	6.76	<0.0001	49.5	50.5	53.9	13.8	0.0697
98	230	222	260	5.21	0.0004	51.1	50.5	53.2	10.2	0.3497
102	219	213	266	7.01	<0.0001	48.3	48.7	54.7	5.81	0.0018
Main effects										
Age					<0.0001					<0.0001
Balanced Protein					0.0100					0.0704
Interaction					<0.0001					0.0072

^1^ S—standard, formulated to meet the nutritional recommendation of Lohmann-LSL guideline; ^2^ L is low, formulated with 20% reduction of balanced protein from S; ^3^ H is high, formulated with 20% increase of balanced protein from S; ^4^ Standard error of the mean.

**Table 8:** Coefficients from linear plus exponential equation for body weight and body composition of laying hens from 8 to 102 weeks old in response to age in three dietary balanced protein levels.

Parameters	Body Weight, g			Ash, g			Fat, g			Protein, g		
	S ^1^	L ^2^	H ^3^	S	L	H	S	L	H	S	L	H
A	1638	1631	1712	46.35	46.35	47.60	345.9	280.0	816.0	240.8	234.0	247.0
B	−2019	−1949	−2176	−68.20	−68.20	−71.57	−382.6	−338.8	−839.0	−347.6	−326.0	−353.7
C	−1.754	−2.569	−0.0916	0.0330	0.0330	0.0641	−1.648	−1.367	−4.780	−0.0775	−0.1303	0.1343
R	0.9164			0.8839			0.9637	0.9468	0.9809	0.9028		
SEM ^4^	78.30			2.670			36.70			15.50		
R^2 5^	91.90			89.50			67.70			87.70		

^1^ S—standard, formulated to meet the nutritional recommendation of Lohmann-LSL guideline; ^2^ L is low, formulated with 20% reduction of balanced protein from S; ^3^ H is high, formulated with 20% increase of balanced protein from S; ^4^ Standard error of the mean; ^5^ Coefficient of determination.

**Table 9:** Egg weight and egg components of laying hens from 19 to 102 weeks old in response to three dietary balanced protein feeds.

Age,	Egg Weight, g					Yolk, %				
Weeks	S ^1^	L ^2^	H ^3^	SEM ^4^	*p*-Value	S	L	H	SEM	*p*-Value
22	51.2	51.3	51.9	0.590	0.7920	21.6	20.9	21.5	0.280	0.2160
26	57.9	55.5	59.4	0.590	0.0010	24.0	23.9	24.3	0.230	0.5230
30	59.3	58.5	60.4	0.590	0.1710	25.1	24.5	25.3	0.200	0.0320
34	61.1	59.7	61.7	0.590	0.1370	25.6	25.5	25.4	0.180	0.8880
38	61.9	60.6	62.2	0.590	0.2640	26.2	26.2	26.3	0.220	0.9790
42	62.4	61.4	63.6	0.590	0.1130	26.5	26.0	26.7	0.230	0.1380
46	62.2	60.2	63.4	0.590	0.0080	27.1	26.5	26.9	0.200	0.1570
50	63.4	60.3	63.7	0.590	0.0010	27.4	27.0	27.0	0.220	0.3500
54	63.8	62.6	64.7	0.590	0.1210	27.4	26.9	27.1	0.210	0.2960
58	63.1	62.1	64.2	0.590	0.1090	27.3	26.6	27.0	0.200	0.0950
62	65.6	64.4	65.3	0.590	0.4560	27.3	26.9	27.5	0.250	0.3580
66	66.6	64.9	66.2	0.590	0.2390	27.6	27.2	27.8	0.260	0.2260
70	65.9	63.0	66.5	0.590	0.0010	27.6	26.4	27.8	0.320	0.0050
74	66.4	62.4	66.1	0.590	<0.0001	27.3	27.2	27.8	0.210	0.2200
78	66.2	62.0	67.5	0.590	<0.0001	27.0	26.6	27.4	0.220	0.0830
82	65.7	61.9	65.5	0.630	0.0000	27.1	27.1	27.7	0.230	0.1250
86	65.5	61.6	66.1	0.590	<0.0001	27.5	27.1	27.7	0.250	0.2540
90	66.9	61.3	67.6	0.590	<0.0001	27.0	26.1	27.5	0.330	0.0200
94	66.9	61.4	65.6	0.630	<0.0001	26.7	26.2	27.1	0.260	0.0910
98	66.9	62.1	66.8	0.590	<0.0001	26.8	26.3	27.1	0.260	0.1400
102	65.8	63.9	66.2	0.590	0.0620	27.1	26.7	27.6	0.310	0.1360
Main effects										
Age					<0.0001					<0.0001
Balanced Protein					0.0010					0.0600
Interaction					<0.0001					0.0590

^1^ S—standard, formulated to meet the nutritional recommendation of Lohmann-LSL guideline; ^2^ L is low, formulated with 20% reduction of balanced protein from S; ^3^ H is high, formulated with 20% increase of balanced protein from S; ^4^ Standard error of the mean.

**Table 10:** Egg components of laying hens from 19 to 102 weeks old in response to three dietary balanced protein feeds.

Age,	Albumen, %					Shell, %				
Weeks	S ^1^	L ^2^	H ^3^	SEM ^4^	*p*-Value	S	L	H	SEM	*p*-Value
22	67.2	68.1	67.2	0.320	0.1280	11.2	11.0	11.3	0.100	0.2160
26	65.6	65.6	65.2	0.280	0.5660	10.5	10.5	10.5	0.100	0.9710
30	64.5	65.4	64.6	0.220	0.0250	10.4	10.1	10.1	0.100	0.0780
34	64.1	64.3	64.4	0.200	0.6040	10.3	10.2	10.2	0.100	0.3500
38	63.6	63.5	63.5	0.220	0.9890	10.2	10.3	10.2	0.100	0.6580
42	63.6	64.1	63.6	0.250	0.4150	9.91	9.92	9.67	0.100	0.0630
46	63.0	63.5	63.3	0.220	0.3620	9.94	10.1	9.87	0.100	0.1510
50	63.0	63.3	63.4	0.230	0.6030	9.59	9.73	9.58	0.100	0.3610
54	62.8	63.4	63.3	0.230	0.2690	9.74	9.71	9.64	0.100	0.6010
58	63.1	63.8	63.4	0.210	0.1160	9.63	9.65	9.65	0.100	0.9820
62	63.2	63.7	63.1	0.270	0.2490	9.54	9.36	9.46	0.100	0.5010
66	62.9	63.4	62.6	0.310	0.2880	9.46	9.49	9.54	0.100	0.8390
70	63.0	63.8	62.7	0.370	0.1240	9.41	9.45	9.49	0.100	0.8170
74	63.4	63.4	63.0	0.230	0.4200	9.28	9.43	9.26	0.100	0.4160
78	63.7	64.1	63.5	0.250	0.2890	9.32	9.28	9.08	0.100	0.2300
82	63.8	63.7	63.1	0.240	0.1560	9.13	9.21	9.15	0.100	0.8210
86	63.5	63.8	63.3	0.260	0.3130	9.06	9.10	9.09	0.100	0.9700
90	64.1	64.7	63.5	0.310	0.0400	8.99	9.2	9.06	0.100	0.3330
94	64.5	64.7	63.9	0.310	0.1980	8.81	9.15	9.05	0.100	0.0980
98	64.5	65.2	64.1	0.340	0.0790	8.72	8.81	8.85	0.100	0.7340
102	64.1	64.5	63.5	0.370	0.2020	8.79	8.76	8.79	0.110	0.9800
Main effects										
Age					<0.0001					<0.0001
Balanced Protein					0.1010					0.8480
Interaction					0.0970					0.1690

^1^ S—standard, formulated to meet the nutritional recommendation of Lohmann-LSL guideline; ^2^ L is low, formulated with 20% reduction of balanced protein from S; ^3^ H is high, formulated with 20% increase of balanced protein from S; ^4^ Standard error of the mean.

**Table 11:** Coefficients for the exponential equation for egg weight and egg components of laying hens from 19 to 102 weeks old in response to age for three dietary balanced protein levels.

Parameters	Egg Weight, g			Yolk, %			Albumen, %			Shell, %		
	S ^1^	L ^2^	H ^3^	S	L	H	S	L	H	S	L	H
A1	66.60	62.50	66.40	27.10			63.6			8.23		
B1	−45.60	−115.0	−56.20	−81.10			234.0			3.72		
R	0.9450	0.8990	0.9340	0.8860			0.8300			0.9830		
SEM ^4^	1.840			0.6540			0.7650			0.2030		
R^2 5^	72.00			72.20			43.50			86.50		

^1^ S—standard, formulated to meet the nutritional recommendation of Lohmann-LSL guideline; ^2^ L is low, formulated with 20% reduction of balanced protein from S; ^3^ H is high, formulated with 20% increase of balanced protein from S; ^4^ Standard error of the mean; ^5^ Coefficient of determination.

## Data Availability

The data presented in this study are available on request from the corresponding author. The data are not publicly available due to privacy.
